# BRIX3000® Papain Gel for Cavity Treatment in the Adult Patient

**DOI:** 10.1155/2021/6624825

**Published:** 2021-06-12

**Authors:** Leonardo Mancini, Alessia Pisaneschi, Vincenzo Mancini, Marino Ginoble, Vincenzo Quinzi, Enrico Marchetti, Giuseppe Marzo

**Affiliations:** ^1^Department of Life, Health and Environmental Sciences, University of L'Aquila, 67100 L'Aquila, Italy; ^2^Private Practice in Via Degli Eroi n.4, 67051 Avezzano, Italy

## Abstract

Tooth decay is a multifactorial disease. Fermentable sugar, host factors, and cariogenic microbial flora are several agents that influence dental structure. In literature, alternative protocols for treating cavities are always of interest in terms of reducing pain and preserving tissue. In this case report, the use of a new gel-based on papain, which has a chemical effect on bacteria and allows the consistency of the altered tissue to be modified, leads to a less traumatic removal of the infected tissue. In this case report, BRIX3000, a gel with papain as its main ingredient, was used to treat an interproximal cavity on an upper premolar of a 35-year-old man frightened of the turbine. After a clinical check of all the systematic conditions and a first view of the oral cavity, the protocol was explained to the patient, and informed consent was obtained. The protocol involved applying the papain gel directly to the cavity, and after 2.5–3 minutes, it was removed. The complete removal of the infected tissue was achieved in three steps. The patient reported no discomfort, and the cavity was completely treated and ready to be restored. In conclusion, BRIX3000 seems to be a good alternative to the turbine in cavity treatment, particularly in patients who have discomfort during traditional treatments and are frightened of the turbine.

## 1. Introduction

Tooth decay is the most widespread disease in the world and has been defined by WHO as a major public health problem globally [1]. The Global Burden of Disease Study 2017 estimated that 2.3 billion people suffer from caries of permanent teeth, and more than 530 million children suffer from caries of primary teeth [2].

Dental caries is a noncommunicable disease (NCD), mediated by the interaction of various factors (physical, biological, environmental, behavioural, and lifestyle-related) that combine to create a degenerative process on the tooth surface with consequent loss of minerals in the hard tissue of the tooth [3]. Dental caries is a multifactorial pathology where all the participating factors increase the risk of developing the disease. There are several risk factors that contribute to the manifestation of tooth decay such as a high component of cariogenic bacteria, inadequate salivary flow, reduced exposure to fluoride, poor or incorrect oral hygiene, incorrect methods of feeding newborns, and poverty [4]. The carious lesion is caused by an imbalance between the continuous processes of demineralization and remineralization that occur naturally on the hard surface of the tooth. When demineralization processes prevail, they lead to a loss of the mineral component of enamel, dentin, or cement, reaching, if not arrested, a point of no return with the appearance of a severe cavitated lesion [5]. Tooth decay is a dynamic disease where several stages and symptoms follow one another. In early tooth decay (noncavitated), usually, there are no symptoms. When tooth decay advances, there is a progressive involvement of the dentine, and if not treated, there is an involvement of the dental pulp, and symptoms appear (tooth pain, infections, and abscesses, or even sepsis) [1]. When dental caries advances and arrives in dentine, the dentist has to remove the involved dentine: it is possible to do this through different methodologies, such as treatment with rotary instruments, manual excavator, air abrasion, sonic abrasion, ultrasonic methods, lasers, and chemo-mechanical methods. Conventional methods of caries removal (treatment with rotary instruments) are commonly associated with pain, discomfort, and fear [6]. Pain is a subjective experience and is a symptom that undermines the patient's physical and mental integrity; moreover, it is closely related to fear, which is the individual's response to a threatening event. Dental anxiety is regarded as a serious health issue worldwide [7]. A dental phobia is a persistent and excessive fear of dental stimuli and procedures that results in avoidance or significant distress with impact on individual's normal routine, social relationships, occupational or school functioning, and social relationships [8]. In DSM 5 (*Diagnostic and Statistical Manual of Mental Disorder*, *Fifth Edition*), dental phobia is classified as a specific-phobia and, more precisely, included in the blood-injection-injury (BII) phobia type [9]; furthermore, some studies suggest that patients with dental phobia often report more anxiety pertaining to other dental stimuli (e.g., the count of a drill and having a tooth extracted) than to blood and injections; indeed, anxiety regarding blood seems to be relatively uncommon or minor in individuals with dental anxiety [10, 11]. Dental anxiety is first and foremost an oral-health problem as it is associated with a lower frequency of dental visits and a higher prevalence of dental caries [12]. Dentists must diligently manage and care with patients who are anxious to receive dental care because this condition affects about one in seven people [13]. In one study, 67 potentially anxious stimuli were brought to the attention of participants, and the image of a “dentist drilling a tooth or molar” was the seventh most anxious stimulus. [14]. In recent years, research has moved towards the search for chemo-mechanical methods to remove dental caries in order to install a positive dental attitude [15]. Moreover, these methods belong to what is now identified as minimally invasive dentistry (MID). Such an approach adopts a philosophy that integrates prevention, remineralization, and minimal intervention for placement and replacement of restorations. The objective is tissue preservation, which means performing treatment with as little tissue loss as possible [16]. Chemo-mechanical cavity preparation is a new method of treatment that involves the chemical softening of carious dentine followed by its removal by gentle excavation [17]. Some studies in the literature have shown that the perceived pain was less when chemo-mechanical removal agents were used to remove caries compared to conventional rotary tools [18]. In other studies, it has emerged that chemo-mechanical caries removal agents have a very slow action compared to mechanical tools; moreover, it seems that they can be used together to completely remove the carious lesion tissue [7]. It has also emerged that the taste and odour of these used agents based on sodium hypochlorite were unpleasant [19]. Through the use of chemical-mechanical methods of caries removal, it is possible to bypass the use of the drill (reducing dental anxiety) but also to preserve a greater amount of healthy tooth tissue. In fact, these materials allow to create a further break in the collagen of the carious lesion to selectively remove the infected dentin while preserving the affected one that can remineralize. [20]. Chemical agents can be classified into two large families: on the one hand, there are those having sodium hypochlorite as main component, on the other those having enzymes. Over the years, there have been several chemical agents produced for the chemo-mechanical removal of dental caries (GK-101 ®/Nmonochloroglycerine, Caridex ®, and Carisolv ®, Papacarie). BRIX3000 was released in 2012 and is a chemical-mechanical agent based on a proteolytic enzyme and papain, which allows the chemical debridement of the collagen present in dentin. The amount of papain used in the product, equal to 3,000 U/mg in a concentration of 10%, together with the capping by means of the Encapsulating Buffer Emulsion technology, would give the gel an ideal pH to ensure that the enzymes are able to perform a proteolysis, on the collagen of decayed dental tissue. The aim of the present study is to describe BRIX3000 as an alternative method to remove an interproximal cavity of an upper premolar tooth in a 35-year-old patient scared of the turbine.

## 2. Case Report

The present protocol was approved by the Ethics Committee of the University of L'Aquila (Document DR206/2013, 16 July 2013). This study follows the principles of Helsinki for human experimentation. This case report was assessed according to the CARE case report guidelines [21]. All the procedures were followed after the signs of the informed consent.

### 2.1. Patient Information

The patient was a 35-year-old nonsmoker who has not been followed by the dentist for some years. We found out through his medical history that he was in good general health and had no significant medical conditions or drug allergies. The man told us that he was afraid of the dentist and the turbine due to bad experiences in the past, and this fear was the cause of poor oral hygiene. He also reported having extracted the second premolar and first molar in the first sextant but could not remember the reason for the extraction. The patient came under our observation after suffering a crown fracture of 2.4 (endodontically treated tooth) and 2.6; he also suffered from dental sensitivity to hot, cold, and sweet stimuli in the days leading up to the visit.

### 2.2. Clinical Findings

During the intraoral visit, it has been found that the patient had poor oral hygiene with dental plaque, and he has been motivated to improve it. There were residual roots of the first premolar and first molar in the second sextant which have been extracted before the cavity's treatment. There was also a Class II interproximal cavity in the mesial tooth wall of the second upper left premolar; on the contrary, there were no fistula and endo-perio lesions.

### 2.3. Timeline

**Figure 1:**
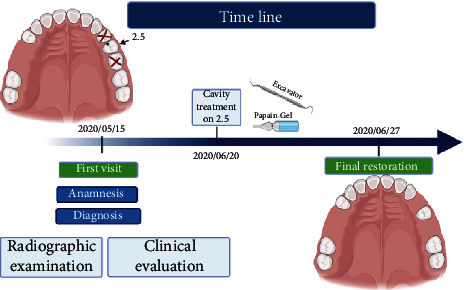
Timeline according to the CARE protocol.

The general health and oral health status of the patient have been identified during the first visit. An intraoral radiography has been made in order to identify the residual roots and diagnose caries. After the extraction of the residual roots of 2.4 and 2.6, a mechanical-chemical removal of the dental cavity has been made allowing to control drill fear of the patient (Figure 1).

### 2.4. Diagnostic Assessment

**Figure 2:**
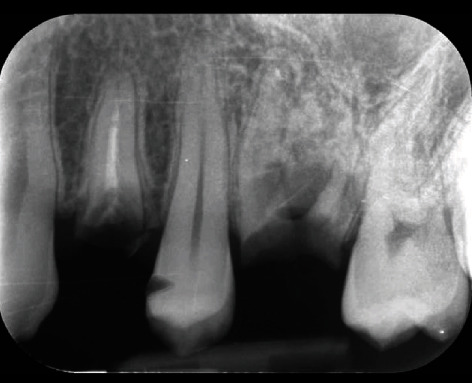
X-ray of the premolar with the presence of residual roots and the cavity on the crown.

The dental cavity in the interproximal level of the second premolar in the mesial wall has been diagnosed through an explorer (3A/6 Explorer, Hu-Friedy Mfg. Co., LLC 3232 N. Rockwell St.; Chicago) and a diagnostic X-ray. This diagnosis confirmed that caries reached the dentine by going beyond the amelo-dentinal junction (Figure 2).

### 2.5. Therapeutic Intervention

**Figure 3:**
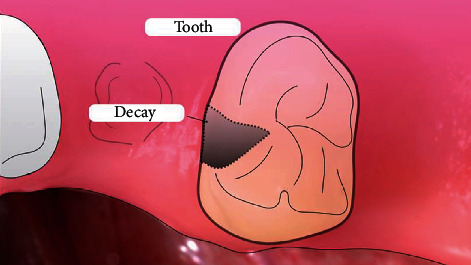
Iconographic representation of the tooth decay on the interproximal surface.

The clinical examination did not reveal the presence of a fistula or endo-perio lesion. Thus, a Class II stage DII according to the last classification from Anusavice et al. [22] interproximal cavity was diagnosed as represented in Figure 3.

**Figure 4:**
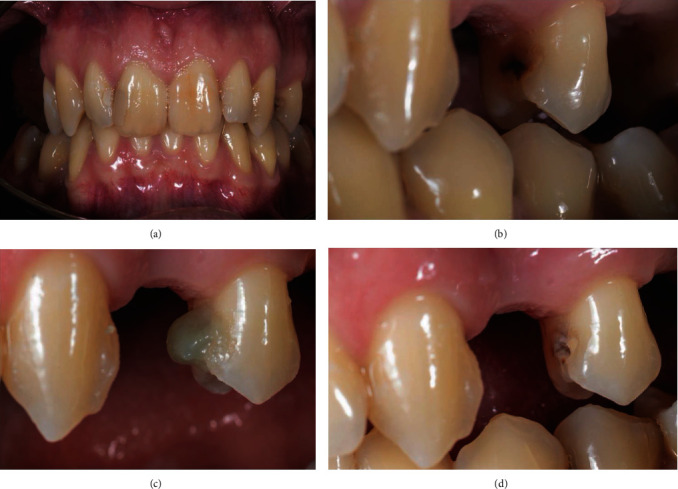
(a) Initial photography. (b) High magnification of the cavity on the second premolar 2.5. (c) First application of BRIX3000. (d) After removing the altered tissue.

The structure around the cavity was sufficient, and the tooth could be treated in a conservative mode, and the missing tissue restored. The man was afraid of dentists and the turbine due to negative experiences in the past. Therefore, a chemo-mechanical removal procedure was selected to reduce the discomfort. The product selected to remove the infected tissue was BRIX3000 papain gel (Brix Medical Science, Carcañá, Argentina). After the informed consent had been signed, the restorative procedure was initiated. The procedure was achieved without anesthesia, and for the first step of the treatment, the isolation was not obtained due to the use of the natural and nondangerous material papain gel. BRIX3000 was applied in the cavity of the 2.5 with a Heidemann spatula (ASA Dental, Bozzano, Italy), and after 2 minutes of activation, following the manufacturer's instructions, the gel started to modify its consistency, producing oxygen bubbles. Its color changed from translucent green to light grey, and at this phase, a dentine excavator (ASA Dental, Bozzano, Italy) was used to remove the altered tissue (Figure 4).

**Figure 5:**
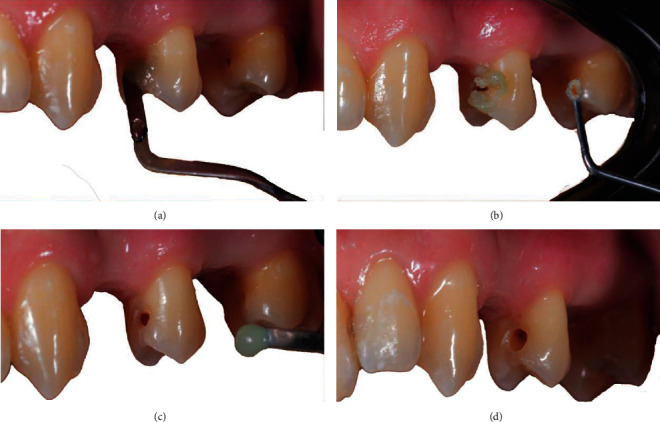
(a) Second application of BRIX3000. (b) The cavity during a second application. (c) Tooth after removing the altered tissue in the second phase. (d) Cavity at the end of the third application. Correction of the profile was performed using the turbine.

The complete removal of the decay was achieved with three applications of BRIX3000. The cavity was completely treated and ready to be restored with a treatment time of 10 minutes (Figure 5).

**Figure 6:**
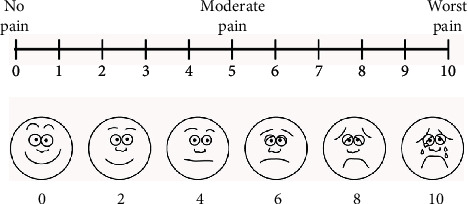
Visual analogue scale to assess pain perception and verify patient discomfort.

During the three steps, a visual analog scale was used (Figure 6). The patient consistently reported a Grade 0 except for the refinement phase, in which a rotary cutting instrument with a spherical cutting tip #014 (Komet Italia Srl, Milano, Italy) was used to modify the profile of the cavity, and Grade 2 was reported.

## 3. Patient Perspective

I received the treatment of the 2.5 premolar using a minimally invasive approach with a substance based on papain gel, and during the therapy, I did not feel disturbed by the treatment preferring the atraumatic remotion to the turbine.

## 4. Discussion

Conventional caries removal techniques are always associated with anxiety and discomfort in children and scared patients. This condition is related to the temperature increasing in the excavation phase which might cause irreversible pulp damage and tooth destruction reducing dentin structure and leading to an impairment of the tooth structure for future restorative therapy [23]. To overcome this condition, several alternative therapies were proposed as laser therapy [24], air abrasion [25], and chemo-mechanical caries removal [26]. Human dentin is composed of water and an organic matrix; the matrix is composed mainly of collagen, chondroitin sulphate, proteoglycans, and phosphoproteins. When carious start to impair the tooth, an acid releasing from several cariogenic bacteria leads to enamel dissolution and progressively in the dentinal tubules [27]. The pH reduction and the persistence presence of bacteria start to demineralize also the dentin which starts to differentiate in two layers, the first one which is the outer layer, and it is in intimate contact with bacteria without any possibilities to remineralized and a second layer where dentine could be remineralized after removing the outer layer [28]. The chemo-mechanical therapy might be more conservative and predictable concerning the traditional rotary instrumentation indeed the action of the papain enzyme is focused only on the outer infected layer where it starts to degrade the collagen of the infected tissue promoting the healthy dentine preservation [29]. The complete cavity treatment respect to the traditional method required more time but with a minimally invasive approach and in a more conservative way. The chemo-mechanical method is a promising alternative to traditional therapy and might be a possible alternative also during the recent issue for the presence of aerosol in the dental office during the COVID-19 outbreak [30, 31]. In this case report, Brix 3000 was used which is the last papain enzyme gel proposed to remove and treat tooth decay. This gel differs from the others for the papain preparation and concentration which might guarantee a better activity on the decay. During the application, the handling on the cavity was easy due to the dried surface which was achieved with a cotton roll. To reduce humidity and ensure a dry environment is possible to use rubber dam that is not tightly required because the gel is natural and in contact with the soft tissue does not arise adverse reactions. Moreover, to the best of our knowledge, the use of this alternative might facilitate, in the future, the approach to the turbine of several scared patients improving their confidence and collaboration for future dental therapies.

## 5. Conclusion

Minimally invasive dentistry is the future of dental practice and requires new materials to be conservative and produce less discomfort for patients. BRIX3000 papain gel seems to be a good alternative in the treatment of tooth decay, reducing the amount of removed tissue and making patients more confident and cooperative with their dentist.

## Data Availability

The data is available at https://app.care-writer.com/public/f8f9f862-0ef5-4d8f-972c-b5f0d843001a.

## References

[1] Gao X., Zhang S. B., Chang Y., Yang F., Zhang Y. (2018). Cryptanalysis of the Quantum Private Comparison Protocol Based on the Entanglement Swapping Between Three-Particle W-Class State and Bell State. *International Journal of Theoretical Physics*.

[2] GBD 2017 Oral Disorders Collaborators, Bernabe E., Marcenes W. (2020). Global, regional, and national levels and trends in burden of oral conditions from 1990 to 2017: a systematic analysis for the Global Burden of Disease 2017 study. *Journal of Dental Research*.

[3] Machiulskiene V., Campus G., Carvalho J. C. (2020). Terminology of dental caries and dental caries management: consensus report of a workshop organized by ORCA and Cariology Research Group of IADR. *Caries Research*.

[4] Selwitz R. H., Ismail A. I., Pitts N. B. (2007). Dental caries. *Lancet*.

[5] Featherstone J. D. B. (2008). Dental caries: a dynamic disease process. *Australian Dental Journal*.

[6] W. H. O. Technical (1944). Sugars and dental caries. *Journal of Public Health Dentistry*.

[7] Yamada T., Kuwano S., Ebisu S., Hayashi M. (2016). Statistical analysis for subjective and objective evaluations of dental drill sounds. *PLoS One*.

[8] Mak C. M., Wong H. M., Xu Y. F. (2011). A four-part setting on examining the anxiety-provoking capacity of the sound of dental equipment. *Noise and Health*.

[9] Seligman L. D., Hovey J. D., Chacon K., Ollendick T. H. (2017). Dental anxiety: an understudied problem in youth. *Clinical Psychology Review*.

[10] (2000). Oral Health in America: A report of the Surgeon General. *Journal of the California Dental Association*.

[11] van Houtem C. M. H. H., Aartman I. H. A., Boomsma D. I., Ligthart L., Visscher C. M., de Jongh A. (2014). Is dental phobia a blood-injection-injury phobia?. *Depression and Anxiety*.

[12] De Jongh A., Bongaarts G., Vermeule I., Visser K., De Vos P., Makkes P. (1998). Blood-injury-injection phobia and dental phobia. *Behaviour Research and Therapy*.

[13] Klingberg G., Berggren U., Carlsson S. G., Noren J. G. (1995). Child dental fear: cause-related factors and clinical effects. *European Journal of Oral Sciences*.

[14] Armfield J. M., Heaton L. J. (2013). Management of fear and anxiety in the dental clinic: a review. *Australian Dental Journal*.

[15] Oosterink F. M. D., De Jongh A., Aartman I. H. A. (2008). What are people afraid of during dental treatment? Anxiety-provoking capacity of 67 stimuli characteristic of the dental setting. *European Journal of Oral Sciences*.

[16] Goyal P. A., Kumari R., Kannan V. P., Madhu S. (2015). Efficacy and tolerance of papain gel with conventional drilling method: a clinico-microbiological study. *Journal of Clinical Pediatric Dentistry*.

[17] Jingarwar M. M., Bajwa N. K., Pathak A. (2014). Minimal intervention dentistry - a new frontier in clinical dentistry. *Journal of clinical and diagnostic research: JCDR*.

[18] Beeley J. A., Yip H. K., Stevenson A. G. (2000). Chemochemical caries removal: a review of the techniques and latest developments. *Nederlands Tijdschrift voor Tandheelkunde*.

[19] Alkhouli M. M., Al Nesser S. F., Bshara N. G., AlMidani A. N., Comisi J. C. (2020). Comparing the efficacies of two chemo-mechanical caries removal agents (2.25% sodium hypochlorite gel and brix 3000), in caries removal and patient cooperation: a randomized controlled clinical trial. *Journal of dentistry*.

[20] Maragakis G. M., Hahn P., Hellwig E. (2001). Clinical evaluation of chemomechanical caries removal in primary molars and its acceptance by patients. *Caries Research*.

[21] Riley D. S., Barber M. S., Kienle G. S. (2017). CARE guidelines for case reports: explanation and elaboration document. *Journal of clinical epidemiology*.

[22] Anusavice K. J. (1992). Decision analysis in restorative dentistry. *Journal of Dental Education*.

[23] Shabzendedar M., Moosavi H., Talbi M., Sharifi M. (2011). Permeability evaluation after decay removal in primary teeth with current caries-excavation techniques. *The Journal of Contemporary Dental Practice*.

[24] Wong Y. J. (2018). Caries removal using lasers. *Evidence-Based Dentistry*.

[25] Banerjee A., Pabari H., Paolinelis G., Thompson I. D., F Watson T. (2011). An in vitro evaluation of selective demineralised enamel removal using bio-active glass air abrasion. *Clinical Oral Investigations*.

[26] Schwendicke F., Paris S., Tu Y. K. (2015). Effects of using different criteria for caries removal: a systematic review and network meta-analysis. *Journal of Dentistry*.

[27] Pitts N. B., Zero D. T., Marsh P. D. (2017). Dental caries. *Nature Reviews Disease Primers*.

[28] Ricucci D., Loghin S., Niu L. N., Tay F. R. (2018). Changes in the radicular pulp-dentine complex in healthy intact teeth and in response to deep caries or restorations: a histological and histobacteriological study. *Journal of Dentistry*.

[29] Júnior Z. S. S., Botta S. B., Ana P. A. (2015). Effect of papain-based gel on type I collagen - spectroscopy applied for microstructural analysis. *Scientific Reports*.

[30] Saccomanno S., Quinzi V., Sarhan S., Laganà D., Marzo G. (2020). Perspectives of tele-orthodontics in the COVID-19 emergency and as a future tool in daily practice. *European journal of paediatric dentistry*.

[31] Izzetti R., Nisi M., Gabriele M., Graziani F. (2020). COVID-19 transmission in dental practice: brief review of preventive measures in Italy. *Journal of Dental Research*.

